# The Role of TLR4 on B Cell Activation and Anti-**β**_2_GPI Antibody Production in the Antiphospholipid Syndrome

**DOI:** 10.1155/2016/1719720

**Published:** 2016-10-27

**Authors:** Si Cheng, Haibo Wang, Hong Zhou

**Affiliations:** ^1^Department of Pediatrics, The Affiliated People's Hospital, Jiangsu University, Zhenjiang, Jiangsu 212002, China; ^2^Department of Laboratory Medicine, Changzheng Hospital, Second Military Medical University, Shanghai 200003, China; ^3^Department of Clinical Laboratory and Hematology, School of Medicine, Jiangsu University, Zhenjiang, Jiangsu 212013, China

## Abstract

High titer of anti-*β*_2_-glycoprotein I antibodies (anti-*β*_2_GPI Ab) plays a pathogenic role in antiphospholipid syndrome (APS). Numerous studies have focused on the pathological mechanism in APS; however, little attention is paid to the immune mechanism of production of anti-*β*_2_GPI antibodies in APS. Our previous study demonstrated that Toll-like receptor 4 (TLR4) plays a vital role in the maturation of bone marrow-derived dendritic cells (BMDCs) from the mice immunized with human *β*_2_-glycoprotein I (*β*_2_GPI). TLR4 is required for the activation of B cells and the production of autoantibody in mice treated with *β*_2_GPI. However, TLR4 provides a third signal for B cell activation and then promotes B cells better receiving signals from both B cell antigen receptor (BCR) and CD40, thus promoting B cell activation, surface molecules expression, anti-*β*_2_GPI Ab production, and cytokines secretion and making B cell functioning like an antigen presenting cell (APC). At the same time, TLR4 also promotes B cells producing antibodies by upregulating the expression of B-cell activating factor (BAFF). In this paper, we aim to review the functions of TLR4 in B cell immune response and antibody production in autoimmune disease APS and try to find a new way for the prevention and treatment of APS.

## 1. Introduction

Antiphospholipid syndrome (APS), both as a primary syndrome and as a syndrome secondary to systemic lupus erythematosus (SLE), is a systemic autoimmune disease defined by recurrent arterial/venous thromboembolic events and/or pregnancy morbidity in the presence of high titer antiphospholipid autoantibodies (aPL) in the plasma of patients [1, 2]. APL-induced thrombosis is not only the major pathological basis and the most prominent clinical manifestation, but also the primary cause of death in APS [3]. Some studies have suggested that APS patients still develop great morbidity and mortality despite receiving repeated anticoagulant therapy [4]. High titer of anti-*β*_2_GPI Ab (anti-*β*_2_-glycoprotein I antibodies) plays a pathogenic role in the APS and increases the risk of thrombosis and recurrent miscarriage in APS patients [5]. Many studies have focused on the pathological mechanism in APS, including the effects of anti-*β*_2_GPI Ab on endothelial cells and the formation of thrombosis and inflammation in platelets. However, little attention is paid to the immune mechanism of production of anti-*β*_2_GPI antibodies in APS. Increasing evidence has demonstrated that APS is mainly caused by T cell hyperactivity and B cell overstimulation, which results in the overproduction of autoantibodies [6]. Here, we summarize the roles of TLR4 in the activation and development of B cells and the production of anti-*β*_2_GPI antibodies in APS.

## 2. *β*_2_GPI and Anti-*β*_2_GPI Antibodies in APS

A large number of studies have shown that aPL, including lupus anticoagulant (LA), anticardiolipin antibodies (aCL), and anti-*β*_2_-glycoprotein I antibodies (anti-*β*_2_GPI), is closely involved in the pathological mechanism of APS [7, 8]. Although previously thought to directly recognize anionic phospholipids, most of these aPL are actually directed against phospholipid-binding proteins. And the main antigenic target of aPL is *β*_2_GPI, which can induce impactful humoral and cellular immune responses [9].

*β*_2_GPI is a protein of approximately 50-kDa and composed of five “sushi” domains, of which domain V mediates the binding of the molecule to anionic phospholipids while domain I seems to be the main target of antibodies associated with an increased risk of thrombosis [10]. Besides, *β*_2_GPI exists at least in two different conformations: a circular plasma conformation in which domain I interacts with domain V and an “activated” open conformation [11, 12]. After the positively charged patch of domain V binding to anionic surfaces, the open conformation is obtained. Thus, *β*_2_GPI exposed the hidden epitopes, especially the cryptic epitope on the domain I, which is recognized by anti-*β*_2_GPI antibodies in the APS [13]. Recently, increasing evidence suggests that misfolded *β*_2_GPI proteins are rescued from degradation and transported to the cell surface without processing to peptides when they associate with the peptide-binding groove of HLA class II molecules in the endoplasmic reticulum (ER) [14, 15]. Jiang et al. [16] demonstrated that the misfolded *β*_2_GPI proteins associated with MHC class II molecules are transported intact to the cell surface without processing to peptides. Furthermore, these complexes efficiently stimulate *β*_2_GPI-specific B cells. These results suggest that misfolded *β*_2_GPI proteins presented on MHC class II molecules can efficiently activate *β*_2_GPI-specific B cells.

Serum anti-*β*_2_GPI antibodies are an independent risk factor for APS. Anti-*β*_2_GPI antibodies, as a member of aPL, are required to bind to the cell surface by interacting with *β*_2_GPI on the cell membrane [17]. Compared with other aPL, such as LA and aCL, anti-*β*_2_GPI antibodies were more closely related to the pathological process in APS. An increasing evidence has demonstrated that a “two-hit hypothesis” has been widely accepted to explain that thrombotic events occur occasionally in spite of the persistent presence of anti-*β*_2_GPI antibodies. The brief content of the hypothesis is that besides the persistence of anti-*β*_2_GPI antibodies is a necessary condition; the APS happens in the presence of an additional “second hit,” such as inflammatory responses [18, 19]. Therefore, production of anti-*β*_2_GPI antibodies is the key factor in the pathogenesis of APS.

## 3. TLR4 in the Immune Mechanism of *β*_2_GPI in APS

**Figure 1:**
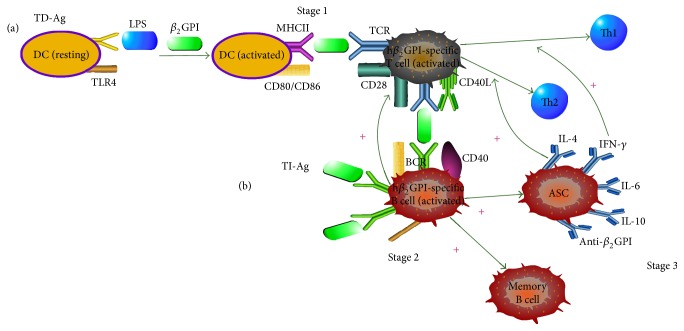
The model for antigen-triggered B cell activation. A model was proposed for B cell-mediated, TLR4-dependent roles of phospholipid-binding protein (human *β*_2_GPI), and innate immune activation in the development of APS-related autoantibodies. This figure outlines two approaches in the process leading to the development of anti-*β*_2_GPI autoantibodies: (a) TD-Ag pathway: Stage 1, activation of DCs and human *β*_2_GPI-specific T cells, Stage 2, activation of human *β*_2_GPI-specific B cells, and Stage 3, the production of anti-*β*_2_GPI autoantibodies and cytokine. (b) TI-Ag pathway: B cell tolerance is broken down in human *β*_2_GPI-specific B cells that recognize self-Ags (human *β*_2_GPI). Then, these B cells present human *β*_2_GPI to any human *β*_2_GPI-specific T cells that can recognize a self-Ag epitope recognized by the B cells, leading to the activation of human *β*_2_GPI-specific T cells. These activated T cells provide help to the cognate B cells, leading to the production of anti-*β*_2_GPI autoantibodies. At last, the activated B cells can present various self-Ag epitopes to T cells with different specificity allowing them in turn to promote the activation of additional human *β*_2_GPI-specific B cells.

A large number of studies have indicated that there is a close relationship between the anti-*β*_2_GPI antibodies and the pathogenic mechanism of APS. Some studies suggested that anti-*β*_2_GPI antibodies were generated by exogenous or endogenous *β*_2_GPI via T cell-dependent or T cell-independent pathways. Thus, we hypothesized a model in Figure 1 to describe the role of TLR4 in the generation of anti-*β*_2_GPI antibodies through T cell-dependent or T cell-independent pathway.

### 3.1. TD-Ag (T Cell-Dependent Antigen)

A mechanism model was proposed for the roles of phospholipid-binding protein and innate immune activation in the development of APS-related autoantibodies. The model contained three stages involving the development of *β*_2_GPI-related autoantibodies. Stage 1 is the activation of dendritic cells (DCs) and human *β*_2_GPI-specific T cells. This step outlines that DCs interact with LPS via its receptor, CD14, leading to TLR4-mediated signaling transduction, APCs activation, and generation of multiple proteins that contribute to inflammation and adaptive immunity. In the presence of human *β*_2_GPI, the activated APCs upregulate the expression of MHC class II and costimulatory molecules CD80/86 and become very effective in presenting human *β*_2_GPI-derived peptide to the human *β*_2_GPI-specific T cells, leading to activation of these T cells. Besides, TLR4-regulated DCs-secreted cytokines influence the development and polarization of Th cells to Th1 or Th2 lineages. Stage 2 is the activation of human *β*_2_GPI-specific B cells. Human CD40L on activated human *β*_2_GPI-specific T cell surface engages its receptor, CD40, on the human *β*_2_GPI-specific B cells, which in turn provide the helper signals for human *β*_2_GPI-specific B cells activation, proliferation, and differentiation into short- and long-lived B cells, such as antibody-secreting plasma cells (ASC) and memory B cells. Stage 3 is the production of anti-*β*_2_GPI autoantibodies and cytokines. At this stage, B cells with high and specific affinity for human *β*_2_GPI-derived peptide differentiate to the human *β*_2_GPI-specific B cells and the antibody-secreting plasma cells produce large amount of anti-*β*_2_GPI autoantibodies as well as cytokines such as IL-4, IL-6, IL-10, and INF-*γ* [20–24].

### 3.2. TI-Ag (T Cell-Independent Antigen)

Chan et al. [25] suggested that the effects of B cells are independent of autoantibody secretion as T cell activation was restored by B cells that could present Ag to T cells and start to secrete Ab. At first, B cell tolerance is broken down in *β*_2_GPI-specific B cells in which endogenous LPS-stimulated, TLR4-mediated signaling transduction is activated. Secondly, these *β*_2_GPI-specific B cells present *β*_2_GPI to any *β*_2_GPI-specific T cells that can recognize an epitope on *β*_2_GPI recognizable B cells. These T cells activate and express CD40L along with other costimulatory molecules and cytokines. Thirdly, these activated *β*_2_GPI-specific T cells facilitate their cognate B cells, leading to increased Ab production, isotype switching, and somatic hypermutation. In this way, multiple autoreactive B cells can be activated by a single human *β*_2_GPI-specific T cells and produce high titer of anti-*β*_2_GPI and other aPL. At last, the activated *β*_2_GPI-specific B cells present *β*_2_GPI epitope to T cells with specificity allowing them in turn to promote the activation of additional autoreactive B cells [26]. This potential mechanism for Ab-independent B cells influenced by T cells is speculated because of the presentation of *β*_2_GPI to T cells. Another potential mechanism is secretion of cytokines, including TNF-a, IL-6, IL-2, and IFN-*γ* [27].

Accumulating evidences demonstrated that B cells have great potential to regulate both innate and adaptive immunity through releasing cytokines. They promote immune responses through Th1/Th2/Th17 and neutrophils, inducing DC maturation, increasing macrophage activation and sustaining antibody production. Moreover, they negatively regulate immune responses by suppressing Th cell responses, inhibiting Th1 cell and Foxp3^+^ Treg differentiation, impairing APC function and proinflammatory cytokines releasing by monocytes, and inducing CD8^+^T cell anergy and CD4^+^ T cell apoptosis [28].

## 4. Signaling Pathway of TLR4 in B Cells in APS

Toll-like receptors (TLRs) are type I transmembrane glycoproteins that function as pattern recognition receptors (PRRs) to recognize a variety of molecules containing pathogen-associated molecular patterns (PAMPs) and/or endogenous damage-associated molecular patterns (DAMPs), leading to the activation of innate immunity. Besides, cytokines are provided with the help of TLRs to induce the differentiation of B cells and T cells, leading to the activation of acquired immunity. Thus, it is believed that TLRs build a bridge between innate immunity and autoimmunity [29, 30]. TLRs are expressed on both lymphoid and nonlymphoid cells including monocytes, macrophages, DCs, B cells, and endothelial cells [31]. However, the first TLR to be recognized is TLR4 [31].

B cells, kinds of acquired immune cells, play a pivotal role in humoral immune response [32]. The expression of TLRs in B cells provides a cell-intrinsic mechanism for innate signals regulating adaptive immune responses [31]. And it has been revealed that TLR4 plays an important role in inflammation [33]. Increasing evidences demonstrated that TLR signaling plays an important role in B cells response-dependent or B cells response-independent T cells. TLR4-mediated B cell activation promotes homing to lymph nodes and localization to germinal centers [32]. The ligation of TLRs can recruit five adaptors: MyD88, TRIF, TIRAP/MAL, TRAM, and SARM. TLR4 activates two main signaling pathways mediated by MyD88 and TRIF, but few studies have examined these pathways in B cells [34].

Barrio et al. [35] investigated the signaling pathways affecting the behaviors of B cells isolated from the spleens of MyD88- or TRIF-deficient mice. They found that 4 h of stimulation with LPS decreased CD69 expression in MyD88^−/−^ B cells, but not in TRIF^−/−^ B cells compared with wild-type (WT) cells. By contrast, LPS stimulation altered the polarization migration and directionality of TRIF^−/−^ B cells and WT cells, but not MyD88^−/−^ B cells. Moreover, LPS stimulation similarly altered both TLR4 and MyD88 signaling pathways. These findings demonstrated that compared with WT B cells, LPS stimulation significantly impaired upregulation of CD86 and proliferation of both MyD88^−/−^ B cells and TRIF^−/−^ B cells and TRIF^−/−^ B cells showed better response than MyD88^−/−^ B cells [36]. These results suggested that TLR4-triggered changes in B cell behaviors including polarization, migration, and directionality were dependent on MyD88 signaling pathway instead of TRIF-mediated signals.

Recently, Janssen et al. [37] confirmed the critical roles of TLR4 in IgE and IgG1 isotype switching in the presence of IL-4 and demonstrated that TRAM/TRIF pathway was essential for IgE isotype switching in mouse B cells. Stimulation with LPS plus IL-4 completely blocked IgE secretion in Tram^−/−^ and Trif^−/−^ B cells. However, stimulation with LPS plus IL-4 reduced IgE secretion in MyD88^−/−^ B cells and IgG1 secretion in Tram^−/−^, Trif^−/−^ and MyD88^−/−^ B cells. Addition of the NF-*κ*B inhibitor, JSH-23, restrained IgE secretion in Trif^−/−^ B cells but had little effect on IgG1 secretion compared to WT B cells. These results demonstrated that constitutive activation of NF-*κ*B driven by TRIF signaling pathway is essential for class switching to IgE in mouse B cells [38]. Thus, MyD88 and TRIF pathways play different roles in regulating TLR4-induced immune responses in B cells.

Zhou et al. [39] found that cognate macrophages, but not T cells, significantly enhanced the B cell activities. Such an enhancement required cell-cell contact. Furthermore, tumor-derived autophagosomes (Dribbles) stimulation upregulated CD40L expression on macrophages, resulting in increased level of CD40 expressed on B cells. The accessory role of macrophages in Dribbles-activated B cells is critically dependent on the CD40/CD40L interaction. In addition, the effects of macrophages were found to be largely dependent on TLR4 and MyD88 signaling pathway. Finally, the results showed that macrophages were able to enhance the antigen presentation function of B cells for specific T cells stimulation.

## 5. The Effects of TLR4 in Promoting the Proliferation, Activation, and Differentiation of B Cells

In our previous studies, we compared the changes of serum anti-*β*_2_GPI antibodies level, B cell activation markers, and related inflammatory molecules as well as spleen germinal centers between *β*_2_GPI-immunized C3H/HeN mice (TLR4 intact) and C3H/HeJ mice (TLR4 defective), to investigate the immune mechanism of Toll-like receptor 4 (TLR4) in the activation of B cells in the spleen of mice immunized with *β*_2_GPI. And the results showed us that the *β*_2_GPI immunization can induce the specific anti-*β*_2_GPI antibodies, TLR4 promotes the production of anti-*β*_2_GPI antibodies, and TLR4 promotes the activation of B cells in *β*_2_GPI-immunized mice. Thus, our results suggest that TLR4 is required for the activation of B cells and the production of anti-*β*_2_GPI antibodies [40].

However, numerous studies mainly focused on the functions of TLR4 in mature B cells. Only few studies have revealed the potential influence of TLR4 on early B cell development. A preliminary report demonstrated that LPS had an inhibitory effect on B lymphopoiesis by promoting myeloid differentiation of hematopoietic progenitors [41]. However, increasing evidences suggest that LPS may promote B cell development and maturation by acting as an accessory complementary to the BAFF physiological pathway [42]. Moreover, LPS, as the natural ligand of TLR4, is known as a potent activator of mature B cells because it improves massive cell polarization and antibody production via the interaction with TLR4 in B cells [43]. IL-7 secreted by bone marrow stromal cells plays a significant role in driving the proliferation of pro-B and pre-B cells. In addition, it may regulate the differentiation of B cell precursors by facilitating the generation of sIgM^+^ immature B cells [44]. Recently, Li et al. [45] confirmed the critical role of TLR4 in the proliferation and differentiation of B cell precursors in TLR4-mutant C3H/HeJ mice and TLR4-intact C3H/HeN mice. LPS-immunized C3H/HeJ mice showed an increase in the number of pro-B and pre-B cells in the bone marrow compared to LPS-immunized C3H/HeN mice. When cultured in the presence of IL-7, the proliferation of pro-B and pre-B cells was significantly inhibited by LPS. In contrast, the generation of IgM^+^/IgD^+^ B cells was greatly enhanced. Overall, these studies suggest that TLR4 signal has a profound influence on the proliferation and differentiation of pro-B and pre-B cells. LPS inhibits IL-7-dependent proliferation of pro-B and pre-B cells and synergizes with IL-7 signals to promote pre-B-cell maturation.

The role of TLR4 in mature B cell activation is well characterized, which can promote the maturation of immature B cells and transitional B cells. Paige et al. [46] reveled that LPS could promote the expression of IgM in B cells and the differentiation of transitional B cells. Moreover, Hayashi et al. [47] explored the effects of TLR4 and TLR2 agonists on B cell development using a model of B cell maturation. B cell maturation was observed in highly purified B220^+^IgM^−^ B cell precursors isolated from C57BL/6 mice by evaluating the expression of IgM, IgD and CD23. LPS stimulation significantly increased the percentage of CD23^+^ B cell precursors. Although Pam3Cys alone had no effect, it restrained LPS-induced increase in population of CD23^+^ B cells. They further investigated the effects of TLR-agonists on early steps of B cell differentiation and found that both lipid A and Pam3Cys impaired IL-7-dependent proliferation, and Pam3Cys treatment retained the precursors on a more immature stage. Taken together, these results suggest that TLR4 signaling plays an important role in B cell development, by promoting the maturation of immature B cells and transitional B cells. In addition, TLR4 signaling favors B lymphocyte maturation, while TLR2 arrests that process.

It is well known that the process of B cells differentiation requires at least two signals. The first one is the recognition of *β*_2_GPI antigen by the B cell-specific receptor (BCR) and the second one is the T cell costimulatory signal for the activation of B cells. The latter is provided by interaction between CD40 in B cells and CD154/CD40L on the surface of activated CD4^+^ T cells. However, increasing evidences indicated that TLRs activation provides a third signal for B cell activation and is significant to antigen-specific antibody responses [48, 49]. TLR4 expression is very low on human B cells surface but increases after stimulation of BCR, CD40, TLRs, and some other cytokines [50]. It is also shown that TLR4 expression is increased on the surface of B cells in peripheral blood of patients with inflammation [51]. Stimulation of B cells via TLR4 not only leads to an increase in antibody production but also alters B cells' other functions such as cytokine production and class-switch recombination and enhances B cell-mediated antigen presentation [52]. During an immune response, B cells can receive signals through both TLRs and BCR. Dual BCR and TLR engagement enhances both innate and adaptive immune functions and further enhances B cell-mediated cytokine and antibody production [53].

Boeglin et al. [54] measured the expression of CD69, CD86, and Blimp-1 mRNA as well as CCL22 production and found that the CD40 pathway synergizes with TLR4 pathways for B cell proliferative response and differentiation into antibody-secreting cells (ASC). Moreover, it is reported that MHC class II-dependent T cell-derived signals are important for B cells in response to T cell-dependent Ag. Bolduc et al. [55] introduced to us a B cell-specific CD40L transgenic mouse model with B cell-restricted MHC class II deficiency and using this model they found that constitutive CD40L expression on B cells alone could not induce differentiation of MHC class II-deficient B cells after immunization with T cell-dependent Ag. Based on above observations, we hypothesized that CD40 pathway and MHC class II synergize with TLR4 pathways for promoting the activation, proliferative, and differentiation of B cells.

## 6. The Effect of BAFF on TLR4-Mediated B Cell Activation

In addition to activation of B cells by BCR, TLR4, and CD40, B cells also receive survival signals via cytokines including B cell activating factor (BAFF). BAFF is a member of the tumor necrosis factor (TNF) families and plays an important role in regulation of B cell survival, proliferation, differentiation, maturation, and immunoglobulin production. It is expressed by innate immune cells such as macrophages/monocytes, DCs, and activated T cells and also by nonlymphoid cells like epithelial cells [56, 57]. Recently, Shen et al. [58] revealed that BAFF could promote the survival/proliferation of mouse splenic B cells. In addition, Yan et al. [59] found a higher BAFF level in hMSCs or mMSCs after TLR4-priming, indicating that TLR4 plays a role in BAFF secretion. Moreover, our previous study found that the expression of BAFF in the spleen from *β*_2_GPI-immunized TLR4 intact mice was significantly higher than that in *β*_2_GPI-immunized TLR4 defective mice [40]. Thus, TLR4 exerts an important function in B lymphocyte-related immune regulation. Overall, TLR4 can promote BAFF expression, thereby promoting B cell activation.

LPS, as a potent B cell mitogen, can promote the activation of TI-Ag induced B cells and the secretion of immunoglobulin. Increasing evidences suggest that TLRs-derived signaling plays a regulatory role in the activation of B cells. However, interaction of BAFF with BAFF-R provides constitutive signals necessary for the development and maturation of B cells [60–62].

In order to investigate the interaction between BAFF and TLR4 in B cells, Hayashi et al. [42] cultured B cells purified from bone marrow in presence with LPS and treated B cell maturation cultures with Fc fusion decoy BAFF-R to block the interaction of BAFF with BAFF-R. They found that the treatment completely abolished the maturation promoted by BAFF but did not inhibit the maturation promoted by LPS, indicating that TLR4 signaling could play an alternative or complementary role to BAFF in B cell development. Moreover, they found that inhibition of NF-*κ*B pathway severely impaired the activity of LPS on the developing B cells in the cultures containing LPS and BAFF, but not BAFF activity. These results lead us to hypothesize that TLR4 can provide, through classical NF-*κ*B activation, maturation, and survival signals alternative or complementary to BAFF.

The immature B lymphocytes undergo an intermediate state before reaching the mature stage, called the “transition” stage. However, maturing B cells exist in the marrow and migrate peripheral lymphatic tissues such as the spleen [63]. The immature B cells of the spleen, called transitional 1 cells (T1), are vulnerable to B cell receptor-dependent cell death. It is reported by Loder et al. [56] that T1 cells lead to another transitional intermediate, the transition 2 (T2) cells. The classical B cell differentiation pathway further demonstrates that marginal zone (MZ) cells and follicular mature (FM) cells are directly developed from T2 B cells with more mature features such as expression of CD23, CD21, and IgD [64, 65]. In addition, it is revealed that transition of from T1 to T2 B cells is dependent on B cell survival and maturation signals generated by the BAFF [66, 67]. The basic signal provided by the interaction between BAFF and BAFF-R is important for the activation of B cells, especially in maturation of T2 and common B cells [68, 69]. Debnath et al. [70] demonstrated that, in BAFF or BAFF-R deficient animal models, peripheral mature B cell populations were significantly decreased, while development of B cells in the bone marrow and migration of T1 B cells into spleen were not changed. In addition, although the overall T2, MZ, and FM B cell populations were decreased in BAFF-R mutant A/WySnJ mice, bone marrow B cell developmental stages and splenic T1 populations were normal, similar to those in BAFF-deficient animals. Other in vitro studies also suggested that TLR4 can promote transformation of B cells precursor in the bone marrow into a transitional B cells with CD23^+^ T2 phenotype [47]. These studies showed that BAFF promoted the transformation of T1 B cells into T2 B cells in the spleen.

BAFF plays an important role not only in the survival, maturation, and differentiation of B cells, but in humoral immunity [69]. Overexpression of BAFF promotes the escaping negative selection of autoreactive T2 B cells, resulting in the malignant tumor of B cells and human autoimmune diseases, such as SLE [71, 72].

## 7. Cytokines Secreted by B Cells

### 7.1. IL-4 Produced by B Cells

IL-4, a cytokine mainly secreted by activated T cells and mast cells, is thought to be important in skewing T cells toward Th2 differentiation and in regulating macrophage proliferation and apoptosis [73]. At present, the studies of B cell-produced IL-4 focus on their importance for Th2 cell development. Nevertheless, it is reported that TLR4 could induce Th2 cell bias polarization [74]. Despite these findings, the role of B cell-derived IL-4 in other types of immune responses needs to be further explored.

### 7.2. IL-6 Produced by B Cells

IL-6, as an important B cell-derived cytokine, was first found in the blood of SLE patients in 1988 [75]. It is reported that interaction of endogenous IL-6 with IL-6R on the surface of SLE B cells led to their terminal differentiation into antibody-secreting cells [76]. Taken together, IL-6 produced by B cells has been implicated in the pathogenesis of autoimmune disease by promoting development of plasma cells.

### 7.3. IL-10 Produced by B Cells

Bhan and his colleagues first introduced the “regulatory B cells” (Bregs) based on their feature of secreting IL-10 in chronic colitis in 1997 and pointed out that Bregs play an important role in autoimmune diseases. Increasing evidences show that the generation of Bregs requires many stimuli mediated signals, such as TLR, BCR, and costimulatory signals, among which TLR signals are the most critical [77, 78]. Murine B cells express most TLR family members; however, whether or not all B cells can develop into Bregs following TLR activation still remains controversial. Mizoguchi and Bhan [79] proposed that only “innate” B cells will develop into Bregs following TLR ligation; others suggested that all immature progenitor B cells have the potential to develop into Bregs after direct stimulation by CD40 and TLR ligation [80]. Emerging evidences suggested that murine B cells can secrete IL-10 after stimulating by both LPS, a TLR4 ligand, and unmethylated CpG dinucleotide, a TLR9 ligand. Some studies reported that mice with B cells lacking TLR4, TLR9, or MyD88 are much more vulnerable to EAE than WT mice and demonstrate increased Th1 and Th17 responses, consistent with the findings observed in B cell-deficient mice or mice with IL-10-deficient B cells. It is believed that IL-10 could inhibit inflammation and autoimmune diseases by various mechanisms in which Bregs were confirmed to play regulatory roles by inducing Tregs, downregulating proinflammatory cytokine production, decreasing MHC II and costimulatory molecule expression, deviating Th cell polarization, and suppressing Th17 cell responses [81–84]. Thus, we proposed that TLR4 can promote B cells to develop into Bregs to secrete IL-10, thus inhibiting the development of inflammation and autoimmune diseases.

### 7.4. IFN-*γ* Produced by B Cells

Accumulating evidences revealed that IFN-*γ*, as an important B cell product, regulates both innate and adaptive immune responses via an autocrine or paracrine manner. In vitro experiments displayed that mouse B cells stimulated by IL-12 could constitutively secrete IFN-*γ*, which then promoted Th1 differentiation through STAT4 activation [85]. Moreover, IL-12-induced IFN-*γ* production by B cells could trigger a series of events in B cells themselves, including STAT1 activation, strong T-bet expression, and IFN-*γ* production via an autocrine manner, leading to Th1-like differentiation [86]. Recent studies showed that B cells may secrete IFN-*γ*, which then elicits various immunoregulatory effects in vivo.

## 8. Targeted Immunotherapy of B Cells in APS

B cells play a significant role in APS and are key players in the development, reactivation, and persistence of autoimmune diseases beyond the production of autoantibodies. B cells are involved in the immune response by producing antibodies and cytokines as well as by their roles in antigen recognition and presentation (independent or dependent of T cells). B cells are also related to a series of aPL correlative clinical events including blocking BAFF, thereby preventing disease occurrence and prolonging survival in APS mouse models.

In the SLE mouse models, BAFF inhibition retained early transitional B cells and B1 cells. The development of B cells is relatively dependent on BAFF because it requires autoantigen recognition and downregulation of BCR. In mice, short-term BAFF blockage modestly decreases the short-lived plasma cells that produce IgM and have no effect on long-lived plasma cells because of the compensation for BAFF deficiency in APRIL signaling through BCMA. After blocking BAFF and APRIL, TACI-Ig significantly reduces short-lived IgM-producing plasma cells and decreases the total number and percentage of IgG-producing plasma cells in the spleen. Multiple intrinsic and adaptive factors may affect the survival of plasma cells in the chronic inflammation and alter their dependence on APRIL and BAFF [87, 88]. In human, compared with IgG-producing plasma cells, IgM-producing and IgA-producing plasma cells are similarly more sensitive to BAFF/APRIL blockage. Some experiments illustrated that survival of murine memory B cells in vivo and of human memory B cells in vitro is independent of BAFF and APRIL signaling. BAFF may reactivate memory B cells in cooperation with inflammatory cytokines and BAFF could play a role in memory B cell function in inflammatory states [89]. In addition, BAFF is essential for the survival of B cells and is involved in many other aspects of B cell biology, including germinal center maintenance, isotype switching, and regulation of B cell-specific markers. Belimumab is an anti-BAFF monoclonal antibody that has reached Phase II trials in SLE and RA, while atacicept (previously known as TACI-Ig), a recombinant fusion protein that neutralizes both BAFF and APRIL (a related B cell survival factor), has undergone Phase I evaluation in SLE [90].

More recently Meroni et al. [91] showed that the subset of anti-*β*_2_GPI autoantibodies specifically reacting against the N-terminal domain (domain I, DI) displays a higher specificity for APS and is a good predictor of thrombosis. They conclude that the antithrombotic effect observed is specifically due to the formation of anti-*β*_2_GPI -DI complexes, which are subsequently cleared from the circulation.

Rituximab, as an anti-CD20 monoclonal antibody, is successfully used in treating patients with rheumatoid arthritis by depleting the immune system of B cells, thus preventing further production of pathogenic autoantibodies. It is associated with a downregulation of aPL titer in addition to its effect on aPL related clinical manifestations and has been shown to reduce the rate of recurrent thrombosis in APS patients [92]. The main objective of rituximab in antiphospholipid syndrome (RITAPS) trial was to evaluate the safety of rituximab in adult APS patients without other systemic autoimmune diseases. The RITAPS trial showed that rituximab in APS patients is safe and that, even without inducing substantial change in aPL, rituximab may effectively control some noncriteria manifestations of aPL [52, 93].

A number of other B cell-directed agents are currently in clinical development. Among the most advanced is epratuzumab, a humanized monoclonal antibody directed against CD22, another B cell-specific marker. Another strategy under investigation is Eculizumab, a humanized monoclonal antibody against complement protein C5, which is a promising future therapy for CAPS [90].

## 9. Conclusion and Prospect

The antiphospholipid syndrome (APS) is characterized by thrombosis and/or recurrent fetal death, associated with the persistence of antiphospholipid antibodies (aPL). In recent years, many studies have focused on the pathological mechanisms of TLR4 in APS, but little attention has been paid to the immune mechanisms of anti-*β*_2_GPI antibodies production in APS. TLR4 promotes the differentiation and migration of B cells via MyD88 pathway, while TRIF signaling pathway is essential to B cells for class switching to IgE. TLR4 not only promotes B cells activation by upregulating BAFF expression in APS but also provides the third signal for B cells activation and for synergization with CD40L and MHC II to promote B cells activation and differentiation into plasma cells to produce anti-*β*_2_GPI antibodies. With the help of TLR4, B cells secrete some cytokines to regulate innate and adaptive immunity in APS autoimmune diseases. In present, there are also numerous drugs targeted to B cells and BAFF for the therapy of APS. However, other TLRs are also expressed in B cells, most of which are involved in the immune response of B cells and could promote B cells activation or differentiation into antibody-secreting cells. For example, TLR2 plays an important role in humoral immunity [94]. Moreover, CD40 can effectively induce the activation, proliferation, and differentiation of resting B cells (RB) that have received first signal via TLR2 [95]. This strategy can be utilized to design vaccines to bolster B cell activation and antigen-presenting efficiency, leading to faster and better immune response in APS.

## Abbreviations

APS: Antiphospholipid syndrome
aPL: Antiphospholipid antibodies
*β*_2_GPI: *β*_2_-glycoprotein I
TLR4: Toll-like receptor 4
BMDCs: Marrow-derived dendritic cells
APCs: Antigen-presenting cells
IL-6: Interleukin 6
IL-10: Interleukin 10
PAMPs: Pathogen-associated molecular patterns
DAMPs: Damage-associated molecular patterns
MHC II: MHC class II molecules.
